# Achilles’ Ear? Inferior Human Short-Term and Recognition Memory in the Auditory Modality

**DOI:** 10.1371/journal.pone.0089914

**Published:** 2014-02-26

**Authors:** James Bigelow, Amy Poremba

**Affiliations:** Department of Psychology, University of Iowa, Iowa City, Iowa, United States of America; Baycrest Hospital, Canada

## Abstract

Studies of the memory capabilities of nonhuman primates have consistently revealed a relative weakness for auditory compared to visual or tactile stimuli: extensive training is required to learn auditory memory tasks, and subjects are only capable of retaining acoustic information for a brief period of time. Whether a parallel deficit exists in human auditory memory remains an outstanding question. In the current study, a short-term memory paradigm was used to test human subjects’ retention of simple auditory, visual, and tactile stimuli that were carefully equated in terms of discriminability, stimulus exposure time, and temporal dynamics. Mean accuracy did not differ significantly among sensory modalities at very short retention intervals (1–4 s). However, at longer retention intervals (8–32 s), accuracy for auditory stimuli fell substantially below that observed for visual and tactile stimuli. In the interest of extending the ecological validity of these findings, a second experiment tested recognition memory for complex, naturalistic stimuli that would likely be encountered in everyday life. Subjects were able to identify all stimuli when retention was not required, however, recognition accuracy following a delay period was again inferior for auditory compared to visual and tactile stimuli. Thus, the outcomes of both experiments provide a human parallel to the pattern of results observed in nonhuman primates. The results are interpreted in light of neuropsychological data from nonhuman primates, which suggest a difference in the degree to which auditory, visual, and tactile memory are mediated by the perirhinal and entorhinal cortices.

## Introduction

It is well established that monkeys’ auditory memory capabilities fall substantially short of their visual and tactile memory capabilities [1], [2], [3], [4], [5]. Many studies have reported that monkeys require extensive training to learn auditory memory tasks [1], [6], [7]. Indeed, some of the earliest attempts to train monkeys on auditory memory tasks reported that subjects learned only “after years of failure”, while others failed to learn at all [1], [8]. Moreover, upon learning the task, they appear capable of retaining auditory information for only a brief period of time. Thus, several experiments have reported that monkeys’ accuracy falls below 75% correct at retention intervals of 40 seconds or less [1], [3], [6]. In contrast, monkeys learn visual and tactile memory tasks relatively quickly and are capable of approximately 75% accuracy at retention intervals of 10 minutes or greater [9], [10], [11]. Inferior memory performance in auditory tasks has been observed in both Old World [3], [6], [7] and New World monkeys [1], [8], as well as in a chimpanzee [12], raising the possibility that auditory memory may be deficient in nonhuman primates in general.

Neuropsychological studies in monkeys suggest that the auditory retention deficit may result, at least in part, from a difference in the degree to which auditory memory is enabled by the perirhinal and entorhinal cortices [4], [6]. While the perirhinal cortex receives substantial projections from visual and tactile cortex, auditory projections are very sparse [4], [13], [14]. Consistent with this anatomical distinction, combined lesions of the rhinal cortices severely disrupt visual and tactile memory [9], [10], [15], but do not significantly impair auditory memory [6]. Moreover, as reported by Fritz et al. [6], visual memory performance of monkeys with combined rhinal lesions is comparable to auditory memory performance of intact monkeys. Thus, auditory memory may not be substantially supported by the rhinal cortices.

While it is clear that auditory memory differs from visual and tactile memory in nonhuman primates, a similar pattern of results has not been clearly established in humans [4], [6], [7]. Many studies conducted over the past century have investigated differences in auditory and visual memory, and some results indicate that humans may be relatively limited in retaining auditory information. For instance, Münsterberg [16] reported over a century ago that subjects were able to recall the serial order of digits and colors with greater accuracy when they were presented visually compared to when they were spoken by the experimenter, also noting that even greater accuracy resulted from combined audiovisual presentation. Similarly, Kirkpatrick [17] found that subjects’ recall for lists of objects was substantially better when they viewed the physical objects themselves compared to when they heard the names of the objects pronounced by the experimenter. This outcome was consistent when subjects’ recall was tested immediately, as well as after a 3-day delay.

Most of the subsequent experiments investigating modality differences have largely concentrated on recall for lists of verbal information such as digits or letters presented in the auditory or visual modalities [18], [19], [20]. Superior accuracy for the visual presentation modality has been observed only when a retention interval follows the list presentation [21]. On the other hand, if subjects are allowed to recall the items from the list immediately after the final item is presented, accuracy is typically higher for the auditory modality [22], primarily due to superior recall of the final items presented in the list (i.e., a greater recency effect).

Beyond recall for lists of verbal cues, Cohen et al. [23] have recently tested subjects’ ability to recognize complex, naturalistic sound clips or images that had been previously presented during a study phase. Recognition accuracy was substantially lower for sound clips than for visual objects, even when additional cues, such as descriptions of the sounds were provided. A subsequent study by Cohen et al. [24] similarly reported inferior auditory recognition memory even in subjects with considerable auditory expertise (professional musicians).

In summary, several experiments using delayed recall and recognition memory paradigms have suggested that humans may have difficulty retaining auditory compared to visual stimuli. However, it is not clear from these studies whether this difference reflects a deficit in auditory compared to both visual and tactile memory (as in nonhuman primates), or whether there might be an advantage for retaining visual over auditory and tactile stimuli. It is also possible that memory might differ for each of these modalities. One study by Larsson and Bäckman [25] provides some evidence that auditory retention may be inferior to both visual and tactile retention. In their study, subjects were briefly exposed to 40 common objects, which were presented in either the auditory, visual, tactile, or olfactory modality. Subjects were then instructed to identify the objects from a list of correct names mixed with distractors. The results indicated that auditory recognition was significantly lower than both tactile and visual recognition, which did not differ from each other. Olfactory recognition was intermediate between auditory and visual/tactile recognition. However, the results of this study were seriously compromised by the fact that the names of the objects were pronounced by the experimenter during the visual, tactile, and olfactory phases (i.e., bimodal presentation), whereas only the name of the object was given during the auditory phase (unimodal presentation). Moreover, subjects were given 6 s to study the objects during the visual, tactile, and olfactory phases, whereas pronouncing the name of the object during the auditory phase was likely accomplished in a shorter amount of time. Thus, it is likely that the bimodal presentation format and longer stimulus exposure time provided as significant advantage for visual, tactile, and olfactory phases compared to the auditory phase.

In addition to these ambiguities, several recent experiments have questioned whether differences reported in human auditory and visual memory tasks reflect inherent mnemonic differences between these sensory modalities [26], [27]. Instead, they have suggested that significant differences in memory functions may result from nonequivalent stimuli or task requirements. For example, Visscher et al. [27] examined auditory and visual short-term memory (STM) using artificial, nonverbal stimuli that had been equated in terms of discriminability, stimulus exposure time, and temporal dynamics. Under these conditions, the decrease in accuracy associated with larger memory sets and longer retention intervals was approximately equal for auditory and visual stimuli. Thus, prior experiments reporting differences in auditory and visual memory might have been biased by differences in discriminability among the stimuli, or perhaps by the verbal nature of the auditory stimuli. It is worth noting, however, that some results reported by Visscher et al. [27] suggested a trend toward a greater recency advantage for auditory stimuli. Because the maximum retention interval used in this study was less than 10 s, it is possible that this trend could become more substantial under more taxing retention demands.

The current experiments were designed to address two primary questions. First, if comparable stimuli are used, are there significant differences in auditory and visual retention capabilities that might emerge at relatively long delays? Second, how might these results compare to tactile memory? Specifically, is there a deficit in auditory memory similar to that reported in nonhuman primate studies? Two experiments tested human subjects’ memory for auditory, visual, and tactile stimuli using STM and recognition memory paradigms. In general, we find support for the hypothesis that auditory memory is inferior to visual and tactile memory.

## Methods

### Experiment 1: Methods

#### Ethics statement

All experiments reported herein were reviewed and approved by the Institutional Review Board at the University of Iowa. All subjects provided informed consent before participating.

#### Subjects

A total of 54 undergraduate students (37 female) with normal or corrected-to-normal vision and hearing participated in this experiment for course credit. All subjects gave verbal consent to participate in the study after reviewing an informed consent document containing details about the study. Written consent was deemed nonessential due to the low-risk nature of the study. All procedures, including the verbal consent process, were approved by the Institutional Review Board at the University of Iowa.

#### Stimuli

The memoranda were simple, non-verbal stimuli that were matched in terms of stimulus exposure time (1 s), temporal dynamics (the stimuli did not vary over time), and discriminability at short retention intervals (described below). Auditory stimuli consisted of pure tones presented binaurally through headphones at approximately 75 dB (HD-280, Sennheiser Electronic Corporation, Old Lyme, CT), visual stimuli consisted of red squares (14 cm) presented against a white background on an LCD monitor positioned approximately 20 cm in front of the subject at eye level (∼38° viewing angle), and tactile stimuli consisted of vibrations presented through a vertical aluminum bar which the subjects gripped with their left hand. The vibrotactile stimuli were produced by passing a digitally-generated sine wave through a tactile transducer (TST209, Clark Synthesis, Inc., Highlands Ranch, CO). The vibrations were generated at a very low intensity to ensure that they were not audible to the subjects (acceleration values measured from the surface of the bar: 0.8±0.1; VM-6360 digital vibration meter, Landtek Instruments, Guangzhou, China). Inaudibility was confirmed with a sound level meter (407740, Extech Instruments Corporation, Nashua, NH), which did not detect change in sound pressure level produced by the vibration stimuli above the ambient noise in the room (35–36 dB).

#### Short-term memory task

Subjects’ STM was tested using the *same*/*different* variation of the delayed matching-to-sample (DMS) task, which is frequently used in testing memory in nonhuman primates [28], [29]. Each trial began with a sample stimulus, which was followed by a variable retention interval of 1, 2, 4, 8, 16, or 32 s, after which a test stimulus that was either identical (same or match trials) or nonidentical (different or nonmatch trials) to the sample. An equal number of match and nonmatch trials using each of the six retention intervals were presented in random order. Upon termination of the test stimulus, the words “Same or different?” appeared on the screen. For match trials, subjects were instructed to click the left button of a mouse held with the right hand, whereas for nonmatch trials they were instructed to click the right button. Following each response, feedback was given by displaying the words “Correct” or “Incorrect” on the monitor for 250 ms, or “No response” if a response did not occur within 1.5 s. “No response” trials were discarded from further analysis (2.0% of total trials). Following feedback, the next trial began after a 1-s intertrial interval (ITI). The experiment was divided into three blocks, each consisting of 12 trials for each retention interval (total = 72 trials per block). Each block was identical except that the modality of the memoranda was either auditory, visual, or tactile. The order in which the sensory modality blocks occurred was fully counterbalanced across subjects, such that nine subjects were randomly assigned to participate in each of the six possible block sequences. All task events were controlled and recorded using E-prime 2.0 (Psychological Software Tools, Inc., Pittsburgh, PA).

Pilot experiments were used to identify two stimulus values for each sensory modality that yielded approximately 90% discrimination accuracy when the stimuli were separated by 1 s. The resulting values were tone frequencies of 1000 and 1018 Hz, red squares with RGB values of 224/0/0 and 255/0/0, and vibration frequencies of 60 and 205 Hz. Within each block of the experiment, the two stimulus values appeared as the sample and test stimuli on an equal number of trials in random order.

### Experiment 2: Method

#### Subjects

A total of 82 undergraduate students (42 female) with normal or corrected-to-normal vision and hearing participated in this experiment for course credit. Subjects gave verbal consent to participate in the study after reviewing an informed consent document containing details about the study. Written consent was deemed nonessential due to the low-risk nature of the study. All procedures, including the verbal consent process, were approved by the Institutional Review Board at the University of Iowa.

#### Stimuli

Using simple, artificial stimuli with carefully controlled stimulus properties in Experiment 1, we observed relatively poor retention of acoustic information compared to visual or tactile information (see Results). The primary goal of Experiment 2 was to investigate the real-world applicability of this finding, i.e., whether this pattern of results generalizes to complex, naturalistic stimuli likely to be encountered in everyday life. Thus, the auditory stimuli used in this experiment were sound recordings of easily-recognizable, everyday events (e.g., dog barking), presented binaurally through headphones (HD-280, Sennheiser Electronic Corporation, Old Lyme, CT). Similarly, visual stimuli comprised silent videos of scenes and events (e.g., scuba diver; dimensions: 6″ × 3.5″, or 15.24 cm × 8.89 cm) presented on an LCD monitor positioned approximately 20 cm in front of the subject at eye level (∼42° viewing angle). For tactile stimuli, common physical objects (e.g., coffee mug) were presented to subjects, which they were allowed to touch and manipulate but not see or hear. A complete list of stimuli used in Experiment 2 is provided in Table S1. During the tactile block, a research assistant sat facing the subject on the opposite side of the desk. The tactile objects were stored on a bookshelf next to the desk, facing away from the subject so that they were not visible. For each trial, the research assistant placed one object inside of an opaque box (48 cm × 55 cm × 33 cm) that was sitting on the desk through an opening in the back of the box (20 cm × 48 cm) that was not visible to the subject. In order to reach the object, the subjects put their arms through two small openings (13 cm × 13 cm) in the front of the box. Heavy tassels hung from the inside of the arm openings to prevent the subjects from seeing the object in the box. Several steps were taken to minimize the possibility that the tactile objects could produce perceptible auditory cues. First, tactile stimuli were initially selected for the experiment on the condition that they did not produce salient or characteristic auditory cues that might reveal the object independent from its physical structure. Second, the box in which the objects were placed was lined with foam to minimize percussive sounds that could be produced when the object was placed inside the box. Finally, the headphones worn by the subjects during the tactile block provided 32 dB of external sound attenuation.

In contrast to the artificial stimuli used in Experiment 1, which can be easily manipulated along a relevant dimension, naturalistic stimuli are much more difficult to control in terms of discriminability and other stimulus attributes. Nevertheless, several measures were taken to ensure that the stimulus sets for each modality were as comparable as possible. First, the stimuli chosen for each sensory modality were temporally dynamic. Thus, videos were chosen as visual stimuli instead of images, because like the naturalistic sound recordings, the stimulus information unfolds over time. Similarly, different parts of the hand and fingers are stimulated over time as subjects touch and manipulate the three-dimensional physical objects, and only partial stimulus information is available to the sensory receptors at a given time.

Second, stimulus exposure time was roughly equated for each modality block. The sound recordings and video clips were each trimmed to 5 s in duration. To ensure that the tactile stimulus exposure time was approximately equal to that of the auditory and visual blocks, cues were presented on the LCD monitor instructing the subjects when to begin and cease touching the objects. During the ITI, a gray screen displayed the words “Put hands in box, but don’t touch object yet” above a countdown starting 5 s before the stimulus presentation period. The screen then turned red and displayed the words “Touch object” above a 5-s countdown indicating the duration of the stimulus presentation period. In addition, subjects wore headphones (Sennheiser HD-280) through which a tone (880 Hz, 500 ms) was presented to signal the beginning of the stimulus presentation period. At the end of the stimulus presentation period, the screen returned to gray for the subsequent ITI countdown or response window depending on whether the stimuli were presented during the study phase or recognition phase (see below).

Finally, before conducting the recognition experiment, 10 subjects (6 female) with native English fluency participated in an object identification task. This was used as a rough index of the discriminability or recognizability of the stimuli for each sensory modality [for a similar approach, see 23]. Each subject was exposed to 100 stimuli for each sensory modality. A single stimulus was presented on each trial, after which subjects were instructed to identify the name of the stimulus from a list of ten options that remained on the screen until a choice was made (chance accuracy = 10%). The nine incorrect object names were randomly selected from the remaining 99 stimuli within the same sensory modality. Following each response, feedback was given by displaying the words “Correct” or “Incorrect” on the monitor along with cumulative accuracy for the session. The feedback display terminated when the subject pressed either of two foot pedals located beneath the desk. Following a 5-s ITI, the next stimulus was presented. Each subject achieved greater than 97% object identification accuracy. For the recognition task, 90 stimuli were selected for each sensory modality block that were correctly identified by all ten subjects (i.e., 100% accuracy).

#### Recognition memory task

The recognition task consisted of a study phase followed by a recognition phase. The study and recognition phases each had separate auditory, visual, and tactile blocks. The order in which the sensory modality blocks occurred was randomized and fully counterbalanced across subjects, such that an equal number of subjects were randomly assigned to participate in each of the six possible block sequences. For each subject, the order in which the sensory modality blocks occurred was the same for both the study and recognition phases. For each block during the study phase, subjects were exposed to 60 stimuli and instructed that their recognition of these items would be tested during the subsequent recognition phase. After each stimulus was presented, subjects were instructed to press either foot pedal to advance to the next stimulus. Stimulus presentations for all blocks were separated by a 5-s ITI to ensure equal temporal spacing of the study items. The recognition phase was similar to the study phase except that 30 of the stimuli for each block were repeated from the study phase (*old* trials) and 30 were presented for the first time (*new* trials). An equal number of *old* and *new* trials were presented in random order, and the stimuli selected for the study and recognition phases were randomized across subjects. Upon termination of each stimulus, the words “Old or new?” appeared on the screen, and subjects were instructed to press the left foot pedal for *new* stimuli and the right foot pedal for *old* stimuli. Following each response, feedback was given by displaying the words “Correct” or “Incorrect” on the screen until a press of either foot pedal initiated the next trial. All task events were controlled and recorded using E-prime 2.0 (Psychological Software Tools, Inc., Pittsburgh, PA).

The time between the study and recognition phases differed for three groups. The *same-day recognition* group (*n* = 24, 11 female) began the recognition phase immediately after the study phase (the study phase lasted approximately 45–60 minutes depending on how quickly the subjects responded and advanced through the directions). The *next-day recognition* group (*n* = 24, 10 female) and *next-week recognition* group (*n* = 24, 15 female) began the recognition phase 24 hours and 7 days after the study phase, respectively.

## Results

### Experiment 1: Results

**Figure 1:**
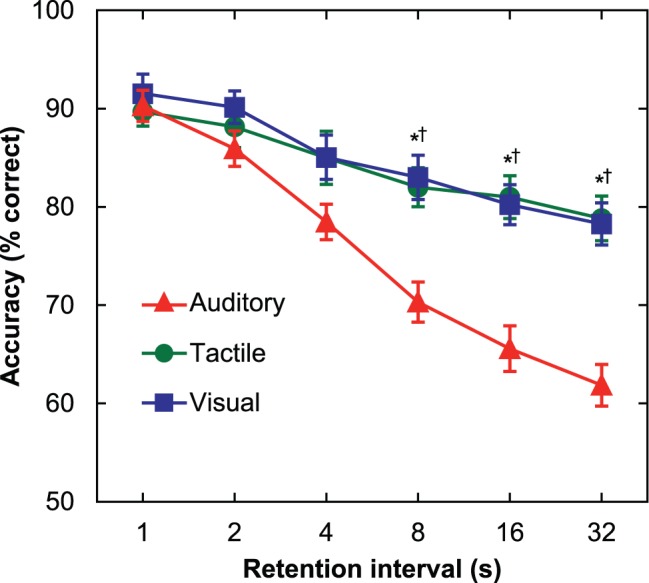
Experiment 1: Mean (± SEM) short-term memory accuracy among sensory modalities for simple, artificial stimuli. Short-term retention of auditory stimuli declines at a greater rate than retention of visual or tactile stimuli. There were no differences in accuracy among the sensory modalities for trials with brief retention intervals (1–4 s), indicating that the initial discriminability of the stimuli was approximately equal. However, at longer retention intervals (8–32 s), accuracy for auditory trials was significantly lower than visual and tactile trials. Post-hoc tests (*p*<.05, Bonferroni correction for multiple comparisons): *Accuracy in the auditory block significantly lower than the tactile block. †Accuracy in the auditory block significantly lower than the visual block.

As seen in Figure 1, accuracy was very similar for each stimulus modality at the 1-s retention interval (auditory: 90.3%; visual: 91.5%; tactile: 89.7%). However, accuracy declined at longer retention intervals to a greater degree for auditory stimuli, such that accuracy at the 32-s retention interval was 61.8%, whereas for visual and tactile stimuli it was 78.3% and 78.8%, respectively. These differences were confirmed by repeated-measures analysis of variance (ANOVA) with sensory modality (auditory, visual, tactile) and retention interval (1, 2, 4, 8, 16, 32 s) as factors, which revealed main effects of both retention interval (*F* [5,265] = 57.88, *p*<.05) and sensory modality (*F* [2,106] = 11.61, *p*<.05), as well as a significant interaction of these factors (*F* [10,530] = 7.78, *p*<.05). Of particular significance, post-hoc tests (*p*<.05; Bonferroni correction for multiple comparisons) revealed that accuracy did not differ among sensory modalities at the 1–4 s retention intervals, suggesting that lower accuracy observed at the longer retention intervals in the auditory block was not attributable to differences in stimulus discriminability.

Two additional analyses were conducted to address the possibility that these results might be attributable to factors other than a deficit in auditory retention capability. First, we investigated whether our results might have been biased by differential practice effects within different sensory modality blocks, similar to those observed in some previous experiments [27]. In other words, it is conceivable that lower mean accuracy in the auditory block could have resulted if the subjects took longer to become familiar with the auditory stimuli than the visual or tactile stimuli. To test this possibility, each modality block of the experiment was subdivided into six successive sub-blocks of 12 trials. Repeated-measures ANOVA with modality and trial sub-block as factors reconfirmed the significant effect of sensory modality block (*F* [2,106] = 11.07, *p*<.05), and indicated that there were significant practice effects (*F* [5,265] = 12.05, *p*<.05). Post-hoc comparisons indicated that subjects improved during the first two sub-blocks of 12 trials, reaching asymptotic performance by the third sub-block. However, there was no significant interaction of sensory modality and trial sub-block (*F* [10,530] = 0.56, *p*>.05), disconfirming the likelihood that the lower mean accuracy observed in the auditory block resulted from slower familiarization with the stimuli.

The second additional analysis was concerned with the potential influence of proactive interference (PI), which may occur if a minimal number of stimuli are recycled as memoranda from trial to trial. Specifically, studies of both human and animal memory show that subjects are more likely to commit an incorrect “match” response on a nonmatch trial if the test stimulus had been presented on the previous trial [29], [30], [31], [32]. In our study, the lower mean accuracy in the auditory block might have been partially influenced by increased susceptibility to PI for auditory stimuli. This possibility was addressed by comparing accuracy on nonmatch trials for which the test stimulus had occurred (PI) or had not occurred (no PI) as the sample stimulus on the previous trial. Repeated-measures ANOVA with modality and PI (PI, no PI) as factors again revealed the significant effect of sensory modality (*F* [2,106] = 3.72, *p*<.05). Contrary to our expectations, however, there was neither a significant effect of PI (*F* [1,53] = 0.01, *p*>.05), nor a significant interaction of PI and modality block (*F* [2,106] = 1.15, *p*>.05). In light of these results, it can be safely concluded that PI did not contribute to the observed performance deficit in auditory trials.

Nonhuman primate studies of memory differences among sensory modalities have typically focused on accuracy and have not reported response latency data. Although Experiment 1 was designed primarily to address possible parallels in human STM to these accuracy differences, response latencies were also analyzed and can be seen in Figure S1. Mean response latency was lower for auditory trials (364 ms) than tactile (385 ms) and visual trials (414 ms), an outcome that echoes the classic finding that simple reaction time is faster when cued by auditory versus visual or tactile stimuli [33], [34], [35]. Repeated ANOVA indicated that the main effect of sensory modality was significant (*F* [2,106] = 8.94, *p*<.05), and post-hoc tests revealed a significant difference between the auditory and visual conditions (*p*<.05; Bonferroni correction for multiple comparisons), but no difference between the auditory and tactile conditions, and only a borderline difference between the visual and tactile conditions (*p* = .051). A significant main effect of retention interval was also observed (*F* [5,265] = 34.43, *p*<.05), where longer retention intervals (and hence, lower accuracy values) were associated with longer response latencies. Unlike the accuracy data, however, the interaction between sensory modality and retention interval was not significant (*F* [10,530] = 1.33, *p*>.05). The absence of a complementary modality/retention interaction in the response latency data likely resulted from several aspects of the task design [36]: (1) subjects were not directed to respond quickly, (2) a generous response window was provided, and (3) subjects were required to wait until the test stimulus had been presented in full before reporting their decision. Future studies may wish to address potential response latency differences using a speeded response design.

In summary, Experiment 1 revealed that retention was limited for auditory stimuli compared to visual or tactile stimuli, even though these stimuli did not differ in terms of discriminability at very short retention intervals. Further analyses revealed that these results were not influenced by differential practice effects or susceptibility to PI among sensory modalities. These results support the hypothesis that, as in nonhuman primates, auditory retention capabilities in humans may be relatively limited.

### Experiment 2: Results

**Figure 2:**
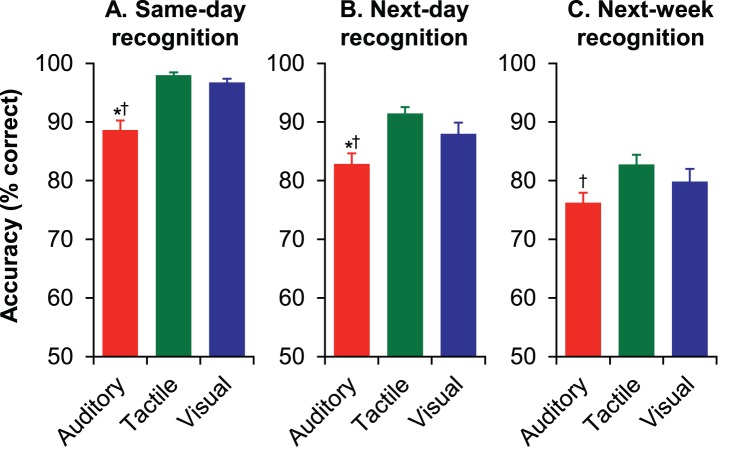
Experiment 2: Mean (+ SEM) recognition accuracy among sensory modalities for complex, naturalistic stimuli. (A) When tested immediately after the study phase, recognition accuracy was lower for auditory stimuli than visual or tactile stimuli. (B) Similarly, recognition was lower for auditory stimuli when tested 24 hours after the study phase. (C) When tested one week after the study phase, recognition accuracy was significantly lower for auditory stimuli than tactile stimuli, but the difference between auditory and visual recognition was not significant. Post-hoc tests (*p*<.05; Bonferroni correction): *Accuracy in the auditory block significantly lower than the tactile block. †Accuracy in the auditory block significantly lower than the visual block.

For the *same-day recognition* group, repeated-measures ANOVA revealed a significant effect of modality block (*F* [2], [46] = 29.69, *p*<.05). Consistent with the pattern of results observed in Experiment 1, post-hoc analyses (*p*<.05; Bonferroni correction for multiple comparisons) indicated that mean accuracy for the auditory block (88.61%) was significantly lower than both the visual (96.74%) and tactile (97.99%) blocks, which did not significantly differ from each other (Figure 2A). Thus both STM for simple, artificial stimuli and recognition memory for complex, naturalistic stimuli appear to be inferior in the auditory modality.

For the *same-day recognition* group, accuracy for both the visual and tactile recognition blocks was near ceiling, which might have concealed differences in recognition memory between these two modalities. For this reason, the *next-day recognition* and *next-week recognition* groups were added to the experiment so that visual and tactile recognition could be compared under conditions in which accuracy was unlikely to reach ceiling. As expected, mean overall accuracy declined at each successively longer delay (one-way ANOVA: *F* [2,69] = 38.61, *p*<.05; all pairwise comparisons significant). Repeated-measures ANOVAs again revealed significant effects of modality block for both the *next-day recognition* group (*F* [2], [46] = 9.51, *p*<.05) and the *next-week recognition* group (*F* [2], [46] = 5.38, *p*<.05). For the *next-day recognition* group, post-hoc comparisons indicated that mean accuracy during the auditory block (82.85%) was again significantly lower than both the visual (87.99%) and tactile (91.46%) blocks, which did not significantly differ from each other (Figure 2B). For the *next-week recognition* group, mean accuracy during the auditory block (76.25%) was significantly lower than the tactile (82.78%) block (Figure 2C). However, although accuracy was lower in the auditory block than in the visual block (79.86%), this difference was not significant. Again, the difference between visual and tactile recognition accuracy was not significant.

Although accuracy predictably decreased with increasing time between the study and recognition phases, as indicated by mean accuracy scores, the magnitude of the deficit in auditory recognition compared to visual and tactile recognition diminished at the longer delays. This outcome contradicted our *a priori* expectation that, since auditory recognition accuracy was relatively poor after a short delay period, this difference would become more pronounced with time. It is also unexpected in light of the sharper decline in accuracy with increasing retention intervals observed during auditory blocks in Experiment 1. Although this trend is somewhat paradoxical, a mixed-factors ANOVA with modality as a within-subjects factor and delay (same day, next day, next week) as a between-subjects factor indicated that the interaction of these variables was not significant (*F* [4,138] = 0.91, *p*>.05). Nevertheless, future studies should be conducted to determine whether a significant trend might emerge using longer delays (and perhaps a fully within-subjects design).

Response latency data for Experiment 2 are presented in Figure S2. Unlike Experiment 1, main effects of sensory modality were not observed in any of the groups (same day: *F* [2], [46] = 2.04, *p*>.05; next day: *F* [2], [46] = 0.96, *p*>.05; next week: *F* [2], [46] = 0.04, *p*>.05). Moreover, although the data trended toward increased response latencies for the longer delays (and lower accuracy values), there was neither a significant main effect of delay (*F* [2,69] = 2.09, *p*>.05) nor an interaction between sensory modality and delay (*F* [4,138] = 0.33, *p*>.05) in a mixed ANOVA with modality as a within-subjects factor and delay as a between-subjects factor. As noted for Experiment 1, this outcome likely reflects the lack of any direction to respond quickly, the requirement that subjects wait until the 5-s stimulus had been presented in full before responding, and the unlimited response window.

In summary, recognition accuracy was lowest for the auditory stimuli in the *same-day recognition*, *next-day recognition*, and *next-week recognition* groups. These differences were statistically significant in nearly all cases, with the exception that auditory accuracy did not differ from visual accuracy in the *next-week recognition* group. Visual and tactile accuracy, on the other hand, did not differ significantly for any of the groups. Together with the results of Experiment 1, these outcomes suggest that, like nonhuman primates, humans are relatively limited in retaining acoustic information.

## Discussion

In general, we observed that retention was inferior for acoustic stimuli compared to visual and tactile stimuli, whereas retention for visual and tactile stimuli was approximately equal. Similar outcomes were observed in tests of STM for simple, artificial stimuli as well as recognition memory for complex, naturalistic stimuli. The deficit in auditory retention was not attributable to differences in the discriminability, exposure time, or temporal dynamics of the stimuli. Further, the results were neither biased by differential practice effects nor by increased susceptibility to PI in the auditory modality.

**Figure 3:**
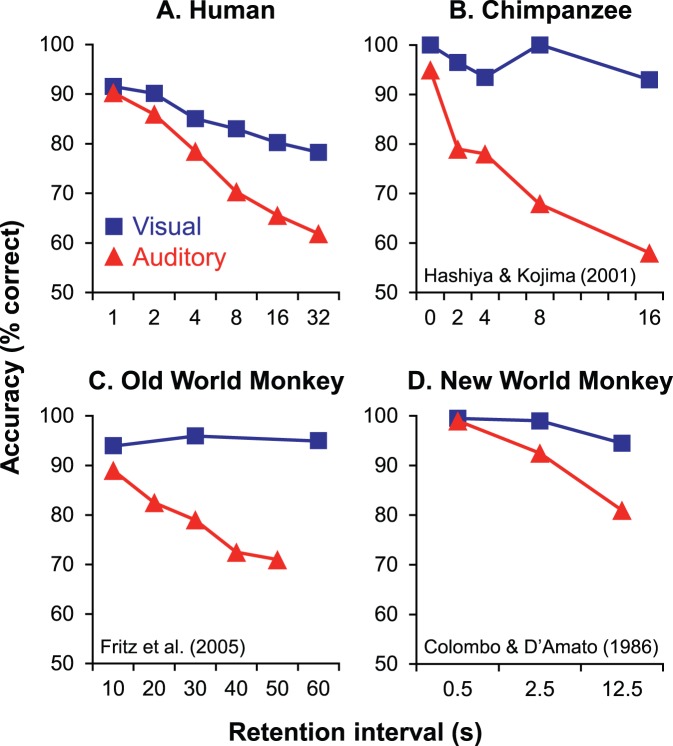
Comparison of visual and auditory short-term memory among primates. (A) In the present experiment, inferior retention was observed for auditory compared to visual stimuli in human subjects. This pattern of results is qualitatively similar to that which has been observed in the chimpanzee (B), as well as both old-world (C) and new-world monkeys (D). (B) Adapted from Hashiya and Kojima [12]; (C) adapted from Fritz et al. [6]; (D) adapted from Colombo and D’Amato [1].

The findings that human STM and recognition memory are inferior for auditory stimuli have several significant implications. In the first place, our results are qualitatively similar to the pattern of results that has been established in the nonhuman primate literature over the past several decades (Figure 3). The findings thus add to the homologies observed between humans and nonhuman primates in numerous other aspects of cognition [37], [38], and importantly, lend increased validity to primate models of human cognitive deficits including amnesia. In addition to these comparative questions, our data strengthen the evidence that memory capabilities are at least in part modality dependent, and thus provide support for theories of memory that account for differences in sensory processing pathways [39].

In nonhuman primates, neuropsychological experiments have suggested that the perirhinal and entorhinal cortices are less involved in auditory memory than visual and tactile memory [4], [6], [9], [10]. Very few studies have addressed whether a similar dissociation might exist in humans. Patients with extensive lesions of the medial temporal lobe, including noted patient H. M., exhibit deficits in both visual and auditory recognition memory [40], [41]. Yet in each of these cases, lesions encompassed not only the perirhinal and entorhinal cortices, but also at least parts of the hippocampus and parahippocampal gyrus. In contrast to the rhinal cortices, the parahippocampal gyrus in nonhuman primates receives significant input from auditory cortices in the superior temporal gyrus [42], [43]. Thus the deficit in auditory recognition may have been caused primarily by damage to the parahippocampal cortex. This suggestion is supported by a human neuroimaging study of auditory and visual recognition memory by Peters et al. [44]. During an encoding session, subjects saw images of common objects presented against a background of either ‘lawn’ or ‘clouds’, and heard names of common objects spoken by either a male or female voice. In the recognition session, visual stimuli were presented on a neutral background and auditory stimuli were spoken by a gender-neutral ‘robot voice’. Subjects were instructed to indicate whether each stimulus was old or new, and for the old items, to report the context in which the item had initially been presented (lawn or cloud background, male or female voice). For auditory but not visual trials, activity in the left and right parahippocampal cortices discriminated between correct and incorrect judgments of the context in which the stimuli had been encoded. On the other hand, overall activation of the right perirhinal cortex was greater during visual encoding, and activity in the left perirhinal cortex discriminated between correct and incorrect context judgments for visual but not auditory trials. The latter observations correspond roughly to the engagement of the nonhuman primate perirhinal cortex in visual but not auditory recognition memory.

It is possible then, that the deficits in auditory retention observed in our experiments as well as in previous studies [23], [24] may reflect a difference in the degree to which memory is supported by the rhinal cortices. If this were true, it would contribute to a growing body of literature suggesting a specialized role for the rhinal cortices in familiarity-based recognition [13], [44], [45]. Indeed, in many of the human and nonhuman primate studies that have reported relatively poor auditory performance (including our own), the tasks are such that successful performance could be accomplished by relying on a familiarity-based recognition strategy. However, additional experiments are needed before this view can be fully validated. For example, human neuroimaging studies using additional stimulus modalities could reveal whether activation of the rhinal cortices is greater during tactile and perhaps olfactory memory compared to auditory memory. In ideal circumstances, studies of patients with lesions restricted to the rhinal cortices could be used to determine whether recognition memory deficits were observed for auditory stimuli. Nonhuman primate studies may also be useful for determining whether parahippocampal lesions might disrupt memory for auditory stimuli, as the studies in humans suggest [44].

Although our findings are consistent with a number of previous human and nonhuman primate studies showing limited retention of auditory information [1], [3], [6], [7], [12], [21], [23], [24], these results do not necessarily imply that memory is inferior in the auditory modality for every taxonomic class of memory. On the contrary, many studies have demonstrated that immediate recall for lists of verbal materials is superior when presented in the auditory modality [18], [19], [20], [21], [22]. Further, lesions that impair familiarity-based forms of recognition memory do not affect other forms of memory such as priming [46]. Thus, future comparisons of memory across sensory modalities should be mindful of specific memory processes likely to be engaged by a given task.

In conclusion, our results suggest that primates may have inferior retention capabilities for auditory events. Further, they imply that memory is to some extent modality dependent, which is likely a consequence of differences among neural pathways in which memoranda are processed. These views are not new; indeed, they have been held by memory researchers for over a century [16], [17], and can be found in folk wisdom dating much earlier. For example, a common English translation of an old Chinese proverb states “I hear, and I forget… I see, and I remember.” In light of the current experimental data, this adage might be amended to include “touch” as an additional mode of superior memory.

## Supporting Information

Figure S1 **Experiment 1: Mean (± SEM) short-term memory response latency among sensory modalities for simple, artificial stimuli.** Longer retention intervals (and hence, lower accuracy values) were associated with longer response latencies, and overall mean response latency was lower for auditory trials (364 ms) than tactile (385 ms) and visual trials (414 ms). However, the interaction between sensory modality and retention interval was not significant. Post-hoc tests (*p*<.05, Bonferroni correction for multiple comparisons): *Accuracy in the auditory block significantly lower than the tactile block. †Accuracy in the auditory block significantly lower than the visual block. ‡Accuracy in the tactile block significantly lower than the visual block.
(PDF)

Figure S2 **Experiment 2:** **Mean (+ SEM) recognition response latency among sensory modalities for complex, naturalistic stimuli.** Significant differences in response latency were not observed among sensory modalities for the (A) same-day, (B) next-day, or (C) next-week recognition delays. Although the data trended toward increased response latencies for the longer delays (and lower accuracy values), there was neither a significant effect of delay nor a significant interaction between sensory modality and delay.
(PDF)

Table S1 **List of stimuli used in Experiment 2.** Auditory stimuli (left two columns) comprised 5-s sound recordings presented through headphones, tactile stimuli (middle two columns) comprised physical objects that the subjects touched and handled, and visual stimuli (right two columns) comprised 5-s silent videos presented on an LCD monitor. Stimulus names are those used for the object identification task described in Methods section of Experiment 2.
(PDF)
