# Supplementary figures and images for: Achilles’ Ear? Inferior Human Short-Term and Recognition Memory in the Auditory Modality
Source: PLoS One. 2014 Feb 26;9(2):e89914. doi: 10.1371/journal.pone.0089914 (PMC3935966; doi:10.1371/journal.pone.0089914)

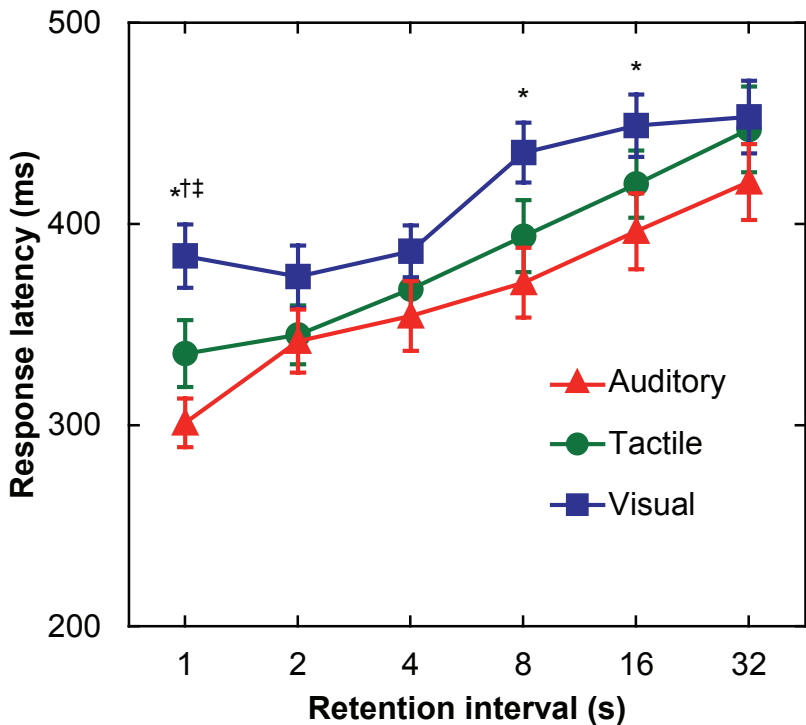

Supplement: Figure S1 — Experiment 1: Mean (± SEM) short-term memory response latency among sensory modalities for simple, artificial stimuli. Longer retention intervals (and hence, lower accuracy values) were associated with longer response latencies, and overall mean response latency was lower for auditory trials (364 ms) than tactile (385 ms) and visual trials (414 ms). However, the interaction between sensory modality and retention interval was not significant. Post-hoc tests (p<.05, Bonferroni correction for multiple comparisons): *Accuracy in the auditory block significantly lower than the tactile block. †Accuracy in the auditory block significantly lower than the visual block. ‡Accuracy in the tactile block significantly lower than the visual block. (PDF) [file pone.0089914.s001.pdf]

**A. Same-day  
recognition**

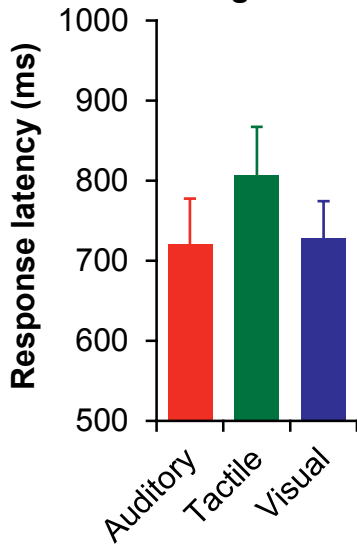

**B. Next-day  
recognition**

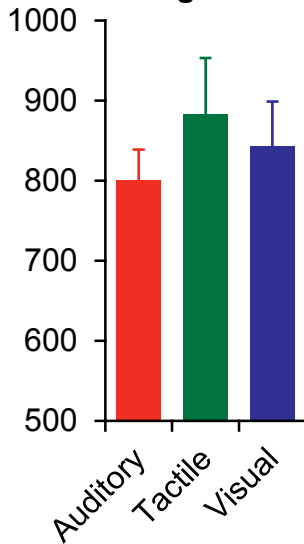

**C. Next-week  
recognition**

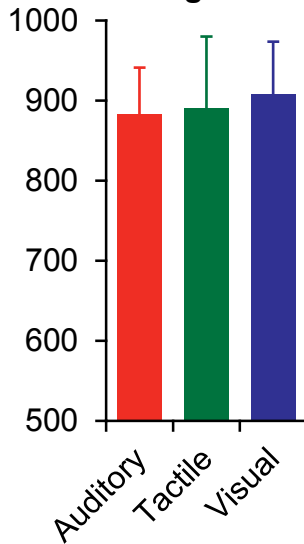

Supplement: Figure S2 — Experiment 2: Mean (+ SEM) recognition response latency among sensory modalities for complex, naturalistic stimuli. Significant differences in response latency were not observed among sensory modalities for the (A) same-day, (B) next-day, or (C) next-week recognition delays. Although the data trended toward increased response latencies for the longer delays (and lower accuracy values), there was neither a significant effect of delay nor a significant interaction between sensory modality and delay. (PDF) [file pone.0089914.s002.pdf]
