# Supplementary material for: Achilles’ Ear? Inferior Human Short-Term and Recognition Memory in the Auditory Modality
Source: PLoS One. 2014 Feb 26;9(2):e89914. doi: 10.1371/journal.pone.0089914 (PMC3935966; doi:10.1371/journal.pone.0089914)
Supplement: Table S1 — List of stimuli used in Experiment 2. Auditory stimuli (left two columns) comprised 5-s sound recordings presented through headphones, tactile stimuli (middle two columns) comprised physical objects that the subjects touched and handled, and visual stimuli (right two columns) comprised 5-s silent videos presented on an LCD monitor. Stimulus names are those used for the object identification task described in Methods section of Experiment 2. (PDF) [file pone.0089914.s003.pdf]

Table S1. List of stimuli[a] used in Experiment 2

Auditory stimuli (sound recordings)		Tactile stimuli (physical objects)		Visual stimuli (silent videos)	
Alarm clock	Gun shots	Action figure	Light bulb	American flag	Jumping a hurdle
Auctioneer	Harmonica	Balloon	Lollipop / sucker	Apples	Karate
Baby crying	Heart beat	Battery	Magazine	Baby	Kite flying
Bagpipes	Heavy breathing	Beads	Magnifying glass	Bald eagle	Ladder
Band instruments	Helicopter	Beer / soda bottle	Marker / highlighter	Basketball	Lifting weights
Banjo	Horse whinny	Belt	Medicine bottle	Beer	Marbles
Bees buzzing	Jack hammer	Bolt	Note pad	Bouncy castle	Microwave
Bells	Laugh	Book	Pacifier	Bow and arrow	Monkey
Bomb dropping	Machine gun	Bowl	Padlock	Brain	Onion
Breaking glass	Maracas	Butter knife	Paint brush	Bread	Orange
Broom sweeping	Motorcycle	Calculator	Paper clip	Cake	Pancakes
Brushing teeth	Opera singer	Camera	Pencil	Camera	Paper airplane
Burp	Piano	Candle	Plastic cup	Cash	Pineapple
Car accident	Pig	Candy bar	Plate	Cement	Police cars
Car starting	Police car sirens	Canned food	Playing cards	Chair spinning	Pond
Cars honking	Pots and pans	Cardboard box	Q tips	Chalkboard	Popcorn
Cash register	Racecar	Cigarette lighter	Rubber band	City	Potato peeling
Cat	Radio tuning	Coat hanger	Scissors	Clock	Printer
Cell phone ring	Saxophone	Coffee mug	Screw driver	Clouds	Ramen noodles
Cell phone vibrating	Sea lion	Coins	Shoe	Cookies	Rat
Chainsaw	Seagulls	Compact disc	Shot glass	Corn	Record spinning
Cheering	Sheep	Computer keyboard	Soda can	Dominos	Rope climbing
Chewing	Siren	Computer mouse	Sponge	Doughnuts	Rubber band ball
Chicken	Snapping	Crayons	Spoon	Drinking fountain	Scuba diver
Coins	Snoring	Credit card	Spray bottle	Drumming	Shaving
Conversation	Soda can	DVD case	Stapler	Ducks	Shooting guns
Coughing	Sword fight	Fingernail clippers	Sticky notes	Earth	Shuffling cards
Cows	Talking on radio	Flash drive	String / Spool of string	Egg	Snake
Crickets	Teapot	Flashlight	Tape dispenser	Eye	Snow shoveling
Crows	Telephone	Flip flop	Telephone	Figure skating	Soccer kick
Cuckoo clock	Throat clearing	Frisbee	Tennis ball	Fire extinguisher	Soda can crushed
DJ turntable scratching	Thunderstorm	Frying pan or pot	Thimble	Fireplace	Space shuttle launch
Dog	Toilet flush	Glasses	Three ring binder	Fire truck	Speedboat
Donkey	Train	Glove	Tissue box	Flash drive	Spider
Door opening	Truck in reverse	Glue bottle	Toilet paper roll	Flower	Stethoscope
Doorbell	Typewriter	Hairbrush	Tongs	Football catch	Swing set
Drumming	Vacuum	Hammer	Toothbrush	Frisbee throw	Toaster
Duct tape	Violin	Hand soap dispenser	Toothpaste	Frog	Tomato
Electric guitar	Water dripping	Hat	Toy car	Golf swing	Toothbrush
Elephant	Whip cracking	Headphones	Toy watch	Guitar	Tractor
Farts	Wind blowing	Ice cube tray	Video game controller	Headphones	Trampoline
Fireworks	Wind chimes	Jar / Jam jar	Wash rag / hand towel	Hockey slap shot	Turtle
Flute	Wolves howling	Ketchup bottle	Water bottle	Horse jumping	Tying shoes
Footsteps	Writing on chalk board	Ketchup packets	Whisk	Hot air balloon	Walk sign
Glass clinking	Yodeling	Legos	Wrench	Jack-o-lantern	Water bottles

[a]Stimulus names are as they appeared in the object identification task
